# Accuracy of robot-assisted implant surgery versus freehand placement: a retrospective clinical study

**DOI:** 10.1186/s40729-024-00586-3

**Published:** 2025-01-03

**Authors:** Hamza Younis, Boya Xu, Kiran Acharya, Longlong He, Liangzhi Du, Sadam Ahmed Elayah, Xiaofeng Chang, Chengpeng Lv

**Affiliations:** 1 Key Laboratory of Shaanxi Province for Craniofacial Precision Medicine Research, College of Stomatology, Xi’an Jiaotong University, Xi’an, China; 2 Clinical Research Center of Shaanxi Province for Dental and Maxillofacial Disease, College of Stomatology, Xi’an Jiaotong University, Xi’an, China; 3 Department of Implant Dentistry, College of Stomatology, Xi’an Jiaotong University, Xi’an, China; 4 Department of Oral and Maxillofacial Surgery, College of Stomatology, Xi’an Jiaotong University, Xi’an, China; 5 State Key Laboratory of Oral Diseases & National Center for Stomatology &, National Clinical Research Center for Oral Diseases, Department of Oral and Maxillofacial Surgery, West China Hospital of Stomatology, Sichuan University, Chengdu, Sichuan China; 6 Department of Implant Dentistry, Xi’an Jiaotong University Hospital of Stomatology, Xi’an Jiaotong University, 98 Xiwu Road, Xincheng Dist, Xi’an, Shaanxi 710004 China

**Keywords:** Accuracy, Computer-aided surgery, Surgical robot, Computer-assisted surgery, Dental implants

## Abstract

**Purpose:**

This study evaluated the accuracy of implant placement using a robotic system (Remebot) compared to freehand surgery and explored factors influencing accuracy.

**Methods:**

This retrospective study included 95 implants placed in 65 patients, divided into robot-assisted (50 implants) and freehand (45 implants) groups. Platform, apical, and angular deviations were measured by superimposing preoperative plans and the postoperative CBCT images. Mean deviations between groups were compared, and regression analysis assessed the impact of implant dimensions and positioning on accuracy.

**Results:**

The robot-assisted group exhibited significantly lower mean deviations in platform (0.44 ± 0.17 mm), apical (0.46 ± 0.17 mm), and angular deviations (0.85 ± 0.47°) compared to the freehand group (1.38 ± 0.77 mm, 1.77 ± 0.82 mm, and 6.63 ± 3.90°, respectively; *p* < 0.001). Regression analysis indicated no significant impact of implant location, jaw type, or implant dimensions on the robotic system’s accuracy, unlike the freehand placement where these factors influenced deviations.

**Conclusions:**

Robot-assisted implant surgery significantly enhances accuracy and clinical safety compared to freehand techniques. Despite limitations, robotic surgery presents a promising advancement in implant dentistry by reducing human error.

## Background

Dental implants represent a highly successful treatment option for restoring the edentulous arch [1]. The precise positioning and angulation of dental implants play a critical role in ensuring their long-term stability. Incorrect placement can lead to adverse tissue healing, potential damage to vital structures, and increased complexity in the subsequent prosthodontic procedures [2].

The application of digital technologies for diagnosis, treatment planning, surgery, and restoration has been lauded as a significant advancement in implant dentistry, offering precise, predictable, and personalized approaches [3]. Computer-assisted implant surgery (CAIS) has effectively reduced the risk of unfavorable outcomes and surgical complications [4]. It has also introduced a higher degree of accuracy and predictability in implant placement when compared to the traditional freehand method [5–7].

Two well-recognized methods within CAIS are static computer-assisted implant surgery (s-CAIS) and dynamic computer-assisted implant surgery (d-CAIS) [8]. The static system employs prefabricated guides to restrict drill movement, directing it precisely to the planned position and angulation during surgery [9, 10]. However, s-CAIS has limitations, including its inflexibility in adapting to changes in the treatment plan, inability to be used in cases with restricted mouth opening, and reduced irrigation and visibility during surgery [11–13]. In contrast, d-CAIS employs active optical tracking to guide the surgeon in three dimensions, using CBCT data to adjust the implant’s position and angulation [14]. This approach offers numerous advantages, such as same-day treatment planning, intraoperative adjustments, adequate irrigation and visibility, and applicability in cases with limited mouth opening [15]. However, using dynamic navigation involves a learning curve and heavily relies on the surgeon’s skill and technique [16, 17]. Nonetheless, both s-CAIS and d-CAIS have demonstrated improved accuracy when compared to freehand implant surgery [14, 18–20].

The recent emergence of robotic computer-assisted implant surgery (r-CAIS) has garnered significant attention in the field of dentistry [21]. This approach combines the advantages of physical constraint seen in s-CAIS with the visibility and flexibility offered by d-CAIS, resulting in a convenient and highly accurate procedure [22]. The FDA-approved YOMI (Neocis Inc, Miami, FL, USA) introduced in 2017, marked the first dental implant surgery robot, employing a passive robotic system that provides haptic and audiovisual guidance during the operation [23]. With YOMI, the surgeon manually guides the robotic arm within the osteotomy site, with the robot constraining hand movements that deviate from the planned path [24].

Several other dental implant robotic systems have been introduced, including task-autonomous robots such as Yekebot (Yekebot Technology Co. Ltd., Beijing, China) and Remebot (Beijing Ruiyibo Technology Co. Ltd., Beijing, China) [25, 26]. In task-autonomous robotic surgery, the surgeon performs pre-surgical preparation, constructs the digital implant plan, and oversees the robot as it independently carries out the implant surgery, intervening only when necessary [21, 23, 26–28].

The advent of robotic systems in implant dentistry represents a significant technological advancement, promising to enhance surgical accuracy and patient outcomes. While the accuracy of implant placement using robotic systems has been evaluated in some studies, clinical and comprehensive comparative analyses with freehand techniques are sparse [22, 26, 29–32]. Clinical studies are imperative as they consider confounding factors such as patient movement and the presence of blood and saliva, which can influence accuracy. This study evaluates the accuracy of implant placement using a robotic system and compares it with freehand surgery.

## Materials and methods

### Study design

This retrospective study evaluated the accuracy of implant placement using a robotic computer-assisted implant system (Remebot, Beijing Ruiyibo Technology Co. Ltd., Beijing, China) and freehand surgery. Records of consecutive patients who received implant surgery, either through robotic-assisted placement or freehand placement, at Xi’an Jiaotong University Hospital of Stomatology (Xi’an, China) between September 2022 and August 2023 were screened and included in this study if they met the inclusion criteria. This research adhered to the Declaration of Helsinki and received approval from the institutional ethics committee at Xian Jiaotong University Hospital of Stomatology, Xian, China (Approval No: xjkqII[2021] No: 043).

The study objectives were as follows:

1. To provide a clinical overview of the utilization of a robotic system in computer-assisted implant surgery.
2. To evaluate the accuracy of implant placement using a robotic surgical system and compare it with that of freehand surgery.
3. To investigate the impact of implant region within the oral cavity on the accuracy of both robot-assisted and freehand surgery.

The inclusion criteria were as follows:

1. Patients over 18 years of age with good general health and oral hygiene.
2. Partially edentulous with at least three remaining teeth in each quadrant, and a maximum of three implants placed in one patient.
3. Adequate alveolar bone width (≥ 6.5 mm) and height (≥ 10 mm) were available at the osteotomy site, which facilitated implant placement using a standard procedure and maintained safety margins with adjacent structures.
4. Written informed consent was obtained from all participants, including consent for the use of the robotic system.

The exclusion criteria were as follows:

1. Heavy smokers (> 10 cigarettes a day).
2. Individuals with uncontrolled systemic diseases, such as diabetes, bleeding disorders, or hypertension.
3. Participants with systemic or local contraindications for implant treatment, such as uncontrolled periodontitis, immunodeficiency, or jaw bone pathologies.
4. Patients with severe alveolar bone defects or those who required alveolar bone augmentation procedures.
5. Patients with missing surgical plans or CBCT data.

All surgical procedures were performed by one skilled and experienced surgeon responsible for preoperative preparation, virtual implant planning, and supervision of the surgical process. The surgeon had undergone comprehensive training in operating the robotic components and software, and had performed numerous surgeries utilizing the robotic system before the commencement of this study. Postoperative CBCT scans were immediately acquired after the surgery. Accuracy was determined by superimposing the postoperative CBCT images onto the preoperative plan. Meyer Dental CBCT equipment (Meyer, China) was employed for all CBCT scans in this study, with the following parameters: a field of view (FOV) measuring 16.7 cm x 11.0 cm, a voxel size of 0.2 mm, and operating parameters set at 100 kV and 10 mA. Implants positioned in the central incisor, lateral incisor, and canine regions were categorized as anterior implants, while those in the premolar and molar regions were considered posterior implants.

### Robot-assisted implant surgery

**Fig. 1:**
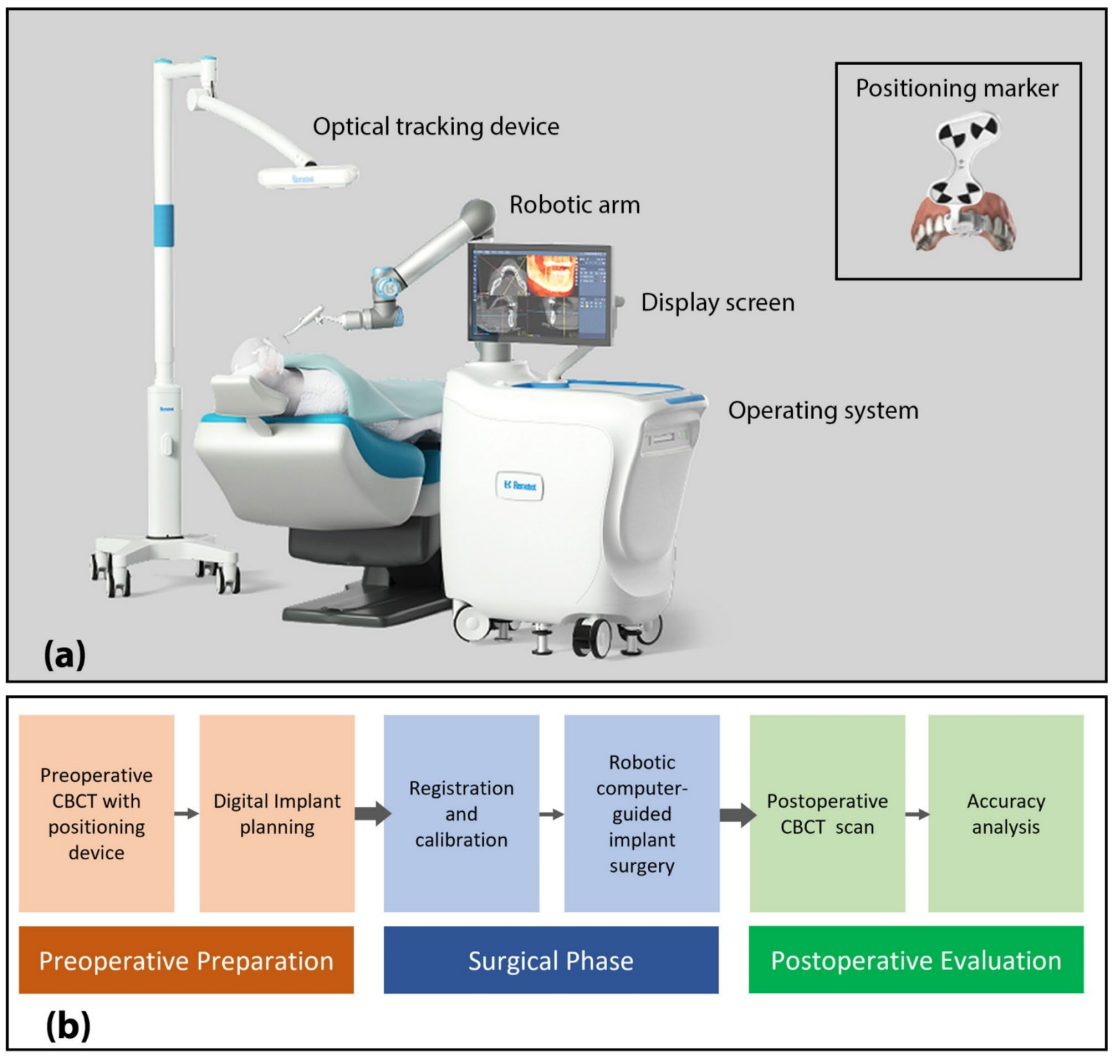
Autonomous implant robotic system (**a**) Components of the robotic system include an operating system, optical tracking device, robotic arm, display screen, and the positioning marker attached to the patient’s jaw. (**B**) Summary of the workflow adopted in the current study

The robotic system comprises a primary unit that includes a robotic arm (UR5, Universal Robots Inc., Odense, Denmark), a display screen, and an operating system (RemebotDent, Beijing Ruiyibo Technology Co. Ltd., Beijing, China). Additional components consist of an optical tracking device (MicronTracker, Claron Technology Inc., Toronto, Canada) and a positioning marker (see Fig. 1a). The treatment protocol, as illustrated in Fig. 1b, encompasses preoperative preparation, the surgical phase, and postoperative assessment.

#### Preoperative planning

**Fig. 2:**
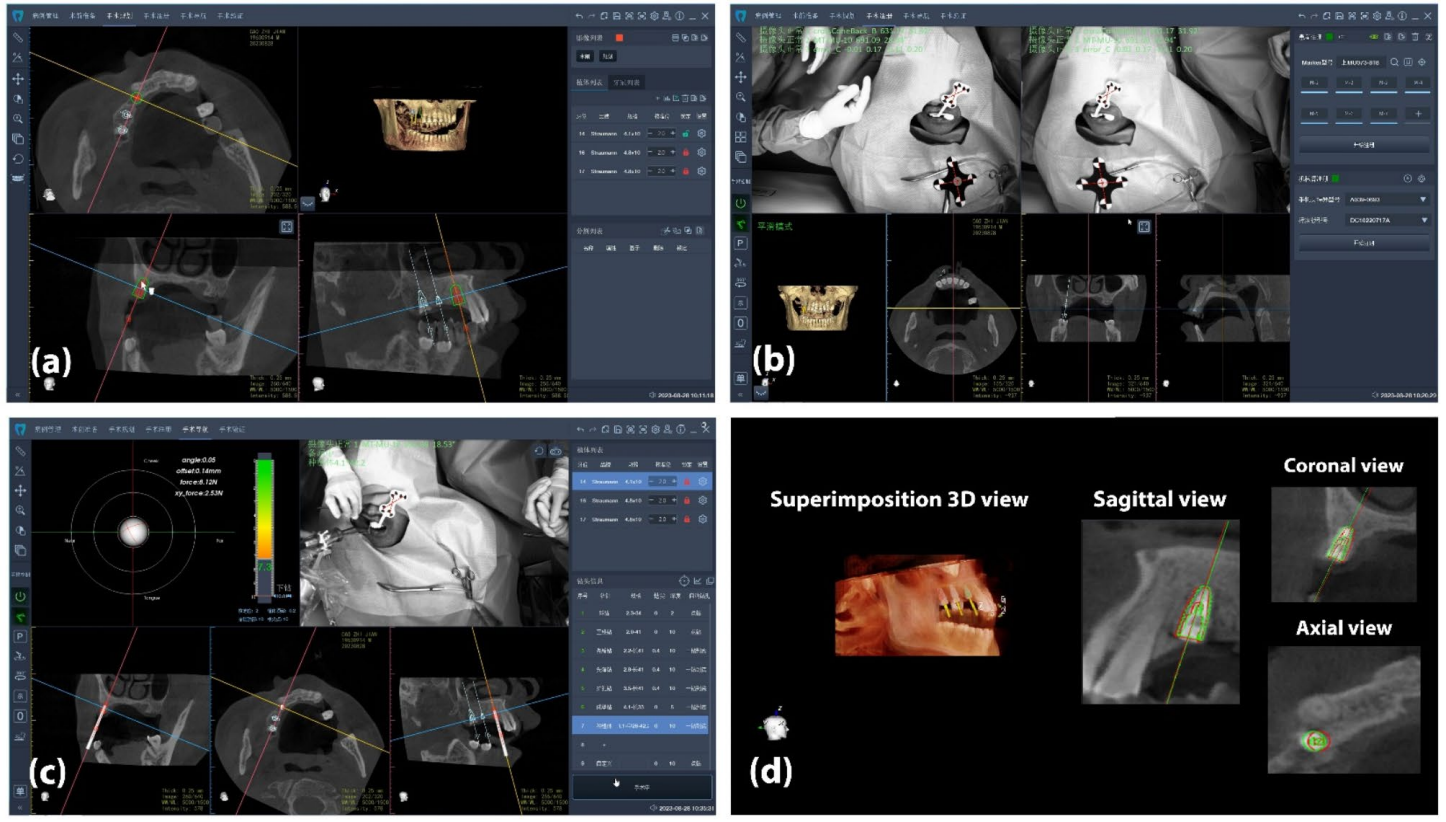
Screenshots depicting the workflow of the autonomous robotic implant surgery (**a**) virtual implant planning performed over the preoperative CBCT (**b**) spatial coordination and calibration between the optical tracker, robotic arm, and oral cavity using optical markers (**c**) autonomous osteotomy preparation by the robot in accordance with the plan with real-time monitoring of the process (**d**) Accuracy evaluation by superimposition, deviation values between the planned (red outline) and placed (green outline) implants were calculated

A positioning device (Beijing Ruiyibo Technology Co. Ltd., Beijing, China) containing radiopaque markers was affixed to the contralateral side of the implant site using self-cured bis-acryl-based resin (Protemp 4, 3 M ESPE, Seefeld, Germany). Subsequently, a preoperative CBCT scan was obtained with the positioning device in place. The CBCT scan was then imported into the RemebotDent software in the digital imaging and communications in medicine (DICOM) format. After segmenting the area of interest, the software automatically detected the markers. The surgeon proceeded to place virtual implants, following a prosthetically-driven approach, and verified the implant specifications and drilling sequence (Fig. 2a). The software recorded the spatial coordinates of the robotic arm and the optical tracking device, and obtained the coordinates of the markers on the positioning device. Ultimately, the software established spatial alignment between the preoperative CBCT, the optical tracking device, and the robotic arm (Fig. 2b).

#### Surgical phase

After extraoral and intraoral disinfection, local anesthesia was administered using 4% Articaine (Primacaine Adrenaline, ACTEON, Merignac, France). After raising a full thickness flap, the surgeon positioned the robotic arm in close proximity to the oral cavity, and calibration was automatically achieved. Subsequently, the robotic arm autonomously carried out the implant osteotomy in accordance with the surgical plan (Fig. 2c). Simultaneously, the surgeon observed real-time feedback data on-screen in various planes and replaced drills as necessary. Finally, a dental implant (BLT, Institut Straumann AG, Basel, Switzerland) was placed autonomously placed by the robot. Post-surgery, patients were provided with oral antibiotics, mouth wash, and analgesics.

### Freehand implant surgery

Patients who underwent freehand implant surgery and had complete data (preoperative CBCT, virtual plan, and postoperative CBCT data) were selected for this study. Before initiating surgery, preoperative CBCT images were uploaded to the RemebotDent software. The surgeon then conducted prosthetically-oriented virtual implant planning to serve as a reference for implant placement and accuracy assessment, and carefully examined the implant’s position, angulation, and proximity to adjacent structures before proceeding with the surgery. Following the administration of local anesthesia, a full-thickness flap was elevated, the surgeon then meticulously examined the osteotomy site, made quick measurements using a periodontal probe, and performed the implant placement as close to the preoperative plan as possible. Finally, a cover screw was affixed to the implant, and the flap was sutured tension-free.

### Postoperative evaluation

**Fig. 3:**
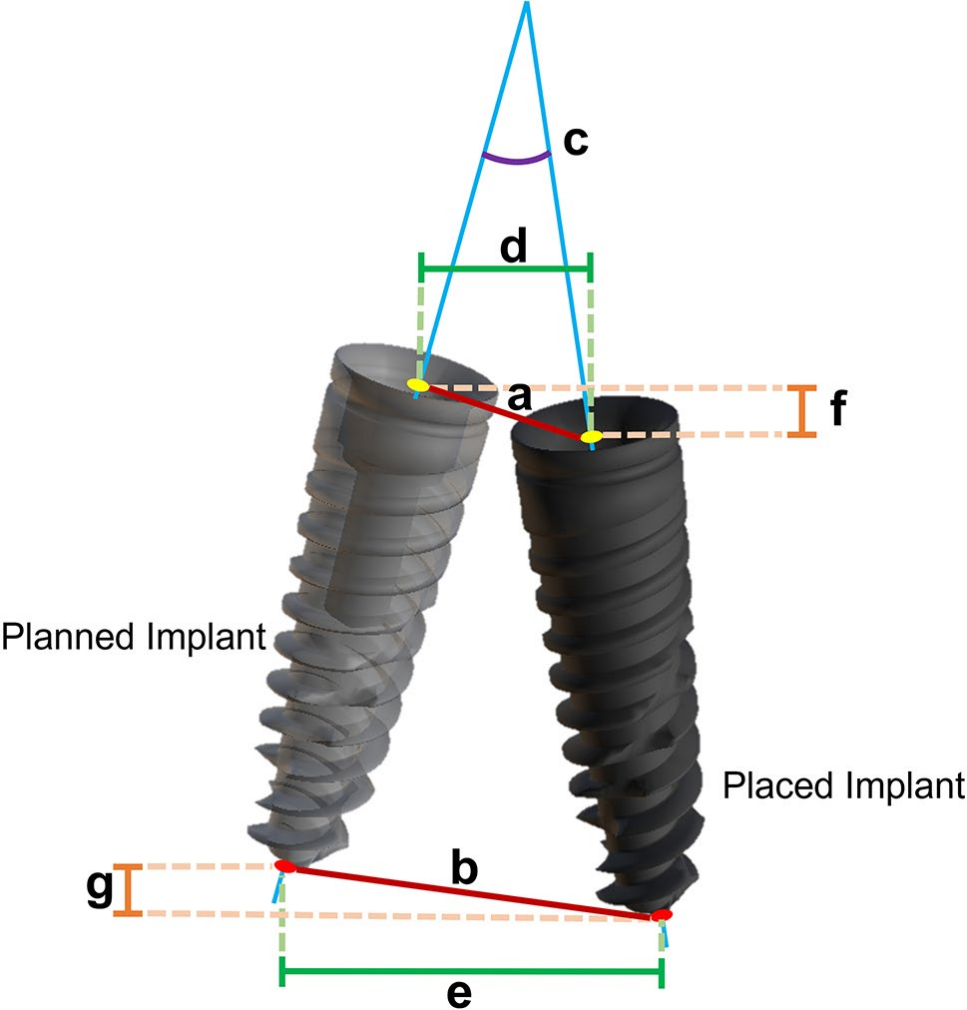
Deviation calculations between the planned and actual implants. (**a**) global platform deviation (**b**) global apical deviation (**c**) angular deviation (**d**) platform lateral deviation (**e**) apical lateral deviation (**f**) platform depth deviation (**g**) apical depth deviation

Both groups shared the same postoperative evaluation process: A postoperative CBCT scan was performed, and DICOM data were transferred to an expert not involved in the treatment for analysis. Patient data were recorded using case numbers without identifiers. The preoperative plan and postoperative data were then uploaded to the RemebotDent software. After superimposition, the software automatically located the implants and calculated the deviation values between the planned and placed implants (Fig. 2d). Finally, the deviation reports were exported and saved. Accuracy data were expressed in terms of deviation values based on the central axes of the planned and actual implants (Fig. 3). Primary outcome variables were global platform, global apical and angular deviations. Lateral and depth deviations at the platform and apex were also calculated. Additionally, subsamples were formed to investigate accuracy variations concerning implant positions, jaw type, and implant dimensions.

For the purpose of statistical analysis, implants were classified into two groups based on their diameter and length. Implant diameters were categorized as “Narrow Diameter” (3.3–3.8 mm) and “Standard Diameter” (4.1–4.8 mm). Implant lengths were categorized as “Standard Length” (8–10 mm) and “Long Length” (12–14 mm). These classifications were used to assess the impact of implant dimensions on the accuracy of implant placement between the robot-assisted and freehand groups.

### Statistical analysis

Statistical analysis was conducted using SPSS^®^ Statistics version 27 (IBM Corp. 2020, NY, USA). Descriptive analysis of accuracy values included means, standard deviations, minimum and maximum values, 95% confidence intervals, and interquartile range. Normal distribution was assessed using the Shapiro-Wilk test, and the equality of variances was examined through Levene’s test. Platform, apical, and angular deviations of the two groups were compared using the independent samples t-test, or the Mann-Whitney U test if data did not exhibit a normal distribution. A multiple linear regression was performed to predict the accuracy of implant placement (platform, apical, and angular deviations) based on jaw type (maxilla vs. mandible), side (left vs. right), position (anterior vs. posterior), implant diameter, and length. The level of significance was set at *p* < 0.05.

## Results

The present study included a total of 95 implants placed in 65 patients. Of these, 50 implants were placed in 35 patients using the Remebot robotic system (Beijing Ruiyibo Technology Co. Ltd., Beijing, China), and 45 implants were placed in 30 patients using freehand surgery. All patients underwent implant surgery at Xi’an Jiaotong University Hospital of Stomatology without any reported adverse events or postoperative complications. Specific patient and implant data are provided in Table 1. All implants were placed in healed sites, with a minimum post-extraction healing period of three months, and each patient received one to three dental implants. The data exhibited a normal distribution.

**Table 1:** Patient and implant distribution

Group	Patients				Implants			
	*n*	Age Range (Mean)	Male/Female		*n*	Left/Right	Maxilla/Mandible	Anterior/Posterior
r-CAIS	35	19–74 (44.49)	14/21		50	25/25	21/29	17/33
Freehand	30	24–68 (47.6)	11/19		45	26/19	28/17	17/28
Total	65	19–74 (45.9)	25/40		95	51/44	49/46	34/61

Abbreviations: r-CAIS = robotic computer-assisted implant surgery

Table 2 provides a summary of the deviation analysis and p-values for both groups. In the r-CAIS group, the mean global platform, global apical, and angular deviations were 0.44 ± 0.17 mm, 0.46 ± 0.17 mm, and 0.85 ± 0.47°, respectively. In the freehand group, the corresponding values were 1.38 ± 0.77 mm, 1.77 ± 0.82 mm, and 6.63 ± 3.90°, respectively. Statistically significant differences (*p* < 0.001) were observed in all seven deviation parameters between the two groups, with r-CAIS exhibiting notably fewer deviations. Significant differences were observed in the platform, apical, and angular deviations between robot-assisted (r-CAIS) and freehand implant placements across all clinical factors evaluated, including jaw type, implant position, side of the arch, implant diameter, and implant length. As shown in Table 3, the r-CAIS group consistently demonstrated significantly lower deviations compared to the freehand group across all parameters.

**Table 2:** Deviation values of r-CAIS and freehand groups

Deviation parameter	Group	Mean ± SD	Median	Min - Max	95% CI	IQR	*p*-value
Global Platform (mm)	r-CAIS	**0.44 ± 0.17**	0.45	0.11–0.79	0.39 to 0.49	0.26	*< 0.001*
	Freehand	**1.38 ± 0.77**	1.24	0.22–3.76	1.15 to 1.62	1.08	
Global Apical (mm)	r-CAIS	**0.46 ± 0.17**	0.46	0.10–0.80	0.41 to 0.51	0.26	*< 0.001*
	Freehand	**1.77 ± 0.82**	1.66	0.39–3.98	1.52 to 2.02	1.19	
Angular (degrees)	r-CAIS	**0.85 ± 0.47**	0.78	0.08–1.88	0.71 to 0.98	0.75	*< 0.001*
	Freehand	**6.63 ± 3.90**	6.32	1.29–18.2	5.46 to 7.80	5.705	
Platform Lateral (mm)	r-CAIS	**0.32 ± 0.14**	0.34	0.07–0.68	0.28 to 0.35	0.19	*< 0.001*
	Freehand	**1.11 ± 0.80**	0.91	0.80–3.68	0.87 to 1.35	1.15	
Apical Lateral (mm)	r-CAIS	**0.33 ± 0.16**	0.32	0.06–0.78	0.29 to 0.38	0.23	*< 0.001*
	Freehand	**1.54 ± 0.87**	1.40	0.27–3.93	1.28 to 1.80	1.21	
Platform Depth (mm)	r-CAIS	**0.27 ± 0.19**	0.28	0.01–0.69	0.22 to 0.32	0.34	*< 0.001*
	Freehand	**0.67 ± 0.45**	0.55	0.05–1.66	0.53 to 0.80	0.62	
Apical Depth (mm)	r-CAIS	**0.27 ± 0.18**	0.28	0.01–0.68	0.22 to 0.32	0.34	*< 0.001*
	Freehand	**0.69 ± 0.46**	0.6	0.03–1.74	0.55 to 0.83	0.65	

Abbreviations: SD = Standard deviation, Min = Minimum, Max = Maximum, CI = Confidence Interval, IQR = Interquartile Range, r-CAIS = robotic computer-assisted implant surgery

**Table 3:** Comparison of platform, apical, and angular deviations between Robot-assisted and Freehand Implant Placement Across various clinical factors

Predictor	Subcategory	Deviation Parameter	*r*-CAIS Mean ± SD	Freehand Mean ± SD	*p*-value
Jaw Type	Maxilla	Platform (mm)	0.41 ± 0.17	1.41 ± 0.84	*< 0.001*
		Apical (mm)	0.42 ± 0.16	1.85 ± 0.93	*< 0.001*
		Angular (deg)	0.75 ± 0.40	8.01 ± 4.12	*< 0.001*
	Mandible	Platform (mm)	0.46 ± 0.18	1.34 ± 0.67	*< 0.001*
		Apical (mm)	0.49 ± 0.18	1.63 ± 0.61	*< 0.001*
		Angular (deg)	0.92 ± 0.51	4.37 ± 2.13	*< 0.001*
Side of arch	Left	Platform (mm)	0.43 ± 0.20	1.23 ± 0.59	*< 0.001*
		Apical (mm)	0.43 ± 0.18	1.57 ± 0.73	*< 0.001*
		Angular (deg)	0.83 ± 0.41	6.50 ± 3.76	*< 0.001*
	Right	Platform (mm)	0.45 ± 0.15	1.59 ± 0.94	*< 0.001*
		Apical (mm)	0.49 ± 0.17	2.04 ± 0.88	*< 0.001*
		Angular (deg)	0.87 ± 0.54	6.81 ± 4.19	*< 0.001*
Position	Anterior	Platform (mm)	0.46 ± 0.14	1.36 ± 0.79	*< 0.001*
		Apical (mm)	0.48 ± 0.15	1.68 ± 0.78	*< 0.001*
		Angular (deg)	0.72 ± 0.46	7.64 ± 4.36	*< 0.001*
	Posterior	Platform (mm)	0.43 ± 0.19	1.40 ± 0.78	*< 0.001*
		Apical (mm)	0.45 ± 0.19	1.82 ± 0.85	*< 0.001*
		Angular (deg)	0.92 ± 0.47	6.02 ± 3.55	*< 0.001*
Implant Diameter	Narrow	Platform (mm)	0.46 ± 0.13	1.20 ± 0.68	*0.008*
		Apical (mm)	0.48 ± 0.15	1.44 ± 0.67	*0.001*
		Angular (deg)	0.68 ± 0.45	5.30 ± 2.14	*< 0.001*
	Standard	Platform (mm)	0.43 ± 0.20	1.44 ± 0.80	*< 0.001*
		Apical (mm)	0.45 ± 0.19	1.86 ± 0.84	*< 0.001*
		Angular (deg)	0.95 ± 0.47	7.02 ± 4.22	*< 0.001*
Implant Length	Standard	Platform (mm)	0.42 ± 0.20	1.33 ± 0.60	*< 0.001*
		Apical (mm)	0.44 ± 0.19	1.75 ± 0.70	*< 0.001*
		Angular (deg)	0.94 ± 0.46	6.43 ± 3.64	*< 0.001*
	Long	Platform (mm)	0.48 ± 0.11	1.62 ± 1.35	*0.048*
		Apical (mm)	0.50 ± 0.12	1.85 ± 1.30	*0.023*
		Angular (deg)	0.65 ± 0.45	7.54 ± 5.20	*0.007*

Abbreviations: r-CAIS = robotic computer-assisted implant surgery, deg = degrees

The multiple linear regression analysis did not show any statistically significant influence of the predictors (jaw type, side, position, implant diameter, and length) on the platform, apical, and angular deviations (*p* > 0.05) in the r-CAIS group. In contrast, in the freehand group, the predictor variables jaw type (*p* < 0.001), implant position (*p* = 0.006), and diameter (*p* < 0.001) significantly influenced angular deviation, with higher angular deviations observed in the maxilla, anterior implants, and implants with a narrower diameter. The results of the multiple linear regression analysis are summarized in Table 4.

**Table 4:** Regression coefficients for platform, apical, and angular deviations of c-CAIS and freehand surgery

Predictor	Deviation parameter	r-CAIS					Freehand			
		B	SE	95% CI	p-value		B	SE	95% CI	p-value
Jaw type (Maxilla vs. mandible)	Platform	-0.18	0.048	-0.113 to 0.076	0.701		-0.112	0.269	-0.656 to 0.433	0.680
	Apical	0.004	0.048	-0.091 to 0.100	0.930		-0.382	0.277	-0.942 to 0.178	0.176
	Angular	0.095	0.159	-0.226 to 0.416	0.553		-4.526	1.015	-6.579 to -2.473	< 0.001
Side of arch (Left vs. right)	Platform	0.038	0.053	-0.069 to 0.146	0.475		0.306	0.239	-0.177 to 0.788	0.208
	Apical	0.094	0.050	-0.007 to 0.195	0.068		0.394	0.245	-0.103 to 0.890	0.117
	Angular	0.119	0.144	-0.172 to 0.409	0.415		0.174	0.899	-1.993 to 1.645	0.848
Position (Anterior vs. posterior)	Platform	0.095	0.137	-0.180 to 0.370	0.491		-0.136	0.341	-0.825 to 0.554	0.693
	Apical	0.060	0.128	-0.198 to 0.319	0.641		-0.074	0.350	-0.783 to 0.635	0.834
	Angular	-0.489	0.368	-1.231 to 0.253	0.191		-3.767	1.285	-6.366 to -1.169	0.006
Implant diameter (Narrow vs. standard)	Platform	0.028	0.139	-0.252 to 0.309	0.840		0.463	0.393	-0.331 to 1.257	0.246
	Apical	0.045	0.131	-0.218 to 0.309	0.731		0.635	0.404	-0.181 to 1.452	0.123
	Angular	0.428	0.375	-0.328 to 1.184	0.260		7.121	1.480	4.128 to 10.115	< 0.001
Implant length (Standard vs. long)	Platform	0.217	0.125	-0.035 to 0.469	0.089		0.314	0.329	-0.351 to 0.979	0.345
	Apical	0.221	0.117	-0.016 to 0.457	0.067		0.120	0.338	-0.563 to 0.804	0.724
	Angular	-0.308	0.337	-0.987 to 0.371	0.365		0.441	1.239	-2.066 to 2.947	0.724

Abbreviations: r-CAIS = robotic computer-assisted implant surgery, SE = Standard Error, 95% CI = 95% Confidence Interval

## Discussion

Accurate positioning of implants is crucial in achieving optimal treatment outcomes and minimizing complications [11]. Placing implants in an unfavorable position has been associated with uneven force distribution, which can lead to failure and damage to the surrounding tissues [33]. Therefore, a method to accurately transfer a prosthetically-oriented implant plan into the patient’s mouth is needed. Robot-assisted implant surgery presents a novel approach and breakthrough in computer-assisted implant surgery, promising high precision and reliability. However, only a few studies have evaluated its accuracy in a clinical setting, with most of them being case reports or case series.

Overall, this study has demonstrated that the robotic system can enhance implant placement accuracy, particularly when compared with freehand surgery. A case-series study involving the same robotic system used in the present study reported mean angular, coronal and apical deviations of 1.11°, 0.74 mm, and 0.73 mm, respectively [26]. This study presented even smaller deviations. Computer-assisted implant surgery has demonstrated higher accuracy than the freehand approach [14, 34]. In our study, we included a freehand surgery group for baseline comparison, as freehand implant surgery is still commonly used. Our comparison showed significantly superior accuracy using r-CAIS in all parameters compared to freehand placement. Most notably, the robotic system achieved outstanding angular accuracy with a mean deviation of 0.85 ± 0.47° compared to 6.63 ± 3.90° in the freehand group. This advantage is particularly beneficial in challenging cases with unfavorable alveolar bone morphology and in the esthetic zone where precise angulation is required.

To better understand the robotic system’s performance in various situations, we investigated the influence of implant location, jaw type, and implant dimensions on implant placement accuracy through regression analysis. None of these predictors demonstrated a significant impact on coronal, apical, or angular deviations. A regression analysis by Chen et al. showed similar results [32], supporting the consistency in implant placement accuracy using the robotic system across different clinical scenarios. In contrast, freehand placement accuracy seemed influenced by jaw type, location, and implant diameter. Based on these outcomes, given that most narrower implants were placed in the anterior region, we can expect higher angular deviations in the anterior maxilla. Therefore, clinicians should carefully consider the surgical approach, especially in the esthetic region, as any deviation could complicate the overall treatment process and compromise esthetic and functional outcomes. The deviation values at the implant’s platform and apex in this study align with those reported in other clinical studies on robot-assisted implant surgery, while the angular deviation was the lowest amongst the studies mentioned [22, 26, 31, 32].

While our study primarily focuses on comparing r-CAIS with freehand implant placement, it’s important to consider how these findings relate to the existing literature on s-CAIS and d-CAIS. Static Computer-Assisted Implant Surgery (s-CAIS) employs prefabricated surgical guides, which offer a high degree of accuracy but lack intraoperative flexibility. This inflexibility can be a limitation in cases where changes to the surgical plan are necessary due to unforeseen anatomical challenges. Moreover, the need for a surgical guide can restrict visibility and irrigation during the procedure, which could impact clinical outcomes, especially in cases with limited mouth opening [35]. Several factors can influence accuracy within the static system, including CBCT scan quality, surgical guide fabrication precision, and achieving a proper fit [36, 37]. Moreover, variables like drilling distance, key length, and sleeve height can also affect accuracy [38].

Dynamic navigation provides real-time guidance and allows for intraoperative adjustments, offering a more adaptable approach compared to s-CAIS. However, as the surgery is performed by the surgeon without physical constraints, deviations may stem from hand tremors and poor technique [29]. Literature suggests that a learning curve is associated with dynamic navigation, emphasizing the need for adequate training and practical experience [14, 39]. Furthermore, the reliance on visual tracking and the need for the surgeon to continuously monitor the system can introduce errors, particularly in less experienced hands [15].

In comparison, Robotic Computer-Assisted Implant Surgery (r-CAIS), as demonstrated in our study, combines the benefits of physical constraint seen in s-CAIS with the flexibility and real-time feedback characteristic of d-CAIS. This system utilizes dynamic navigation guidance to autonomously manipulate the robotic arm, aligning with the preoperative plan, and stopping automatically when reaching the planned depth. The robotic system reduces human error stemming from limited visualization and fatigue by providing consistent precision and real-time corrections during the procedure, potentially offering superior accuracy, especially in complex cases [40].

The robotic system appears to provide more accurate implant placement compared to s-CAIS and d-CAIS. Previous studies have shown similar accuracy levels for s-CAIS and d-CAIS [2, 41]. Although there are currently no formal randomized controlled trials on r-CAIS, the results of this study and available literature consistently support the enhanced accuracy of r-CAIS. According to previous meta-analyses, the mean angular, coronal, and apical deviations for s-CAIS ranged between 3.5–5.3°, 0.99–1.11 mm, and 1.24–1.6 mm, respectively, while for d-CAIS the corresponding values ranged between 3.68–3.8°, 0.81–1.03 mm, and 0.91–1.33 mm, respectively [7, 35, 42–45]. A recent in vitro study comparing r-CAIS and d-CAIS reported mean coronal, apical, and angular deviations of 0.46 ± 0.29 vs. 0.70 ± 0.30 mm, 0.56 ± 0.30 vs. 0.85 ± 0.25 mm, and 1.36 ± 0.54 vs. 1.80° ± 0.70°, respectively, significantly lower for r-CAIS [28].

**Fig. 4:**
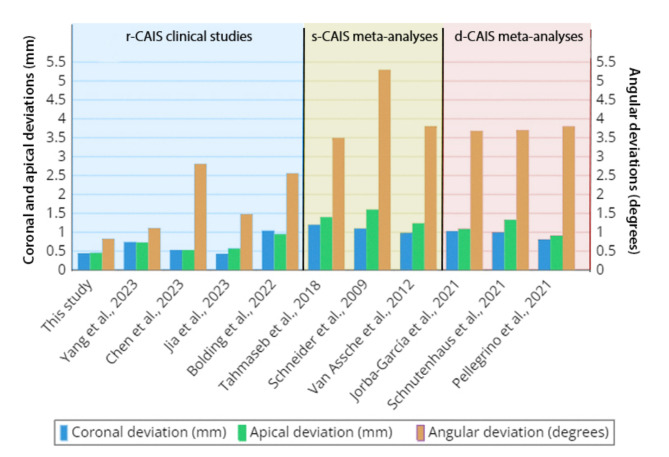
Mean deviations comparison between the present study and other studies involving r-CAIS, s-CAIS, and d-CAIS

In addition to the comparative analysis between r-CAIS and freehand surgery, we have included Fig. 4, which presents the accuracy data of r-CAIS, s-CAIS, and d-CAIS techniques from previous studies, focusing on coronal, apical, and angular deviations [7, 35, 42–45]. The data demonstrate that r-CAIS consistently shows lower deviations across all three parameters—coronal, apical, and angular—compared to both s-CAIS and d-CAIS. This suggests that r-CAIS not only outperforms freehand surgery but also offers superior precision over other computer-assisted methods. The potential of robotic systems to achieve a higher degree of accuracy is evident, particularly in complex cases where precision is critical, such as in the esthetic zone or when dealing with limited bone volume. These insights underscore the potential for robotic technology to set a new standard in implant accuracy, supporting the broader adoption of r-CAIS not only for its precision but also as a means of enhancing overall patient care.

Despite the remarkable performance of robot-assisted implant surgery, several challenges persist. The current robotic system’s high cost and space requirements limit its reliability, accessibility, and cost-effectiveness. The surgical duration is extended due to the necessary preoperative setup and calibration process. The system also requires a consistently clear line of sight between the optical tracker and the surgical site, with unobstructed positioning devices. Any deviation may occur if the robotic arm is not promptly adjusted to the patient’s movements [46]. Additionally, executing implant surgeries in the posterior region with limited mouth opening can be challenging, as the robotic arm adheres to an autonomous path. On an ethical and legal front, although the system has received approval, the relevant ethical and regulatory frameworks might not keep pace with the rapid advancements in r-CAIS technology [47]. The robotic system implements a CBCT-based planning and surgical navigation, making factors like resolution, voxel size, field of view, and discrepancies between the CBCT and the patient’s anatomy critical to accuracy [3]. The quality of CBCT scans and the absence of artifacts are therefore paramount to achieving precision.

The primary limitation of this study lies in its retrospective nature and the absence of s-CAIS and d-CAIS comparison groups. Further prospective studies are essential to compare r-CAIS with s-CAIS and d-CAIS. Potential confounding factors, such as implant distribution and inaccuracies in accuracy assessment (e.g., superimposition errors and CBCT artifacts), are present. The accuracy analysis software provided by the manufacturer relies on the superimposition of preoperative and postoperative CBCT scans, therefore, a postoperative CBCT scan was necessary. Using intraoral scans for accuracy assessment could eliminate radiographic inaccuracies and minimize unnecessary radiation exposure for patients [48]. In essence, this study sought to assess the general performance of the robotic system and provide a baseline comparison with freehand surgery. Our objective was to set path for subsequent research and pinpoint areas where r-CAIS requires validation and further development, especially in complex cases involving guided bone regeneration or sinus floor elevation techniques. Moreover, as this study focused on partially edentulous arches with delayed implant placement, the application of r-CAIS in completely edentulous patients and immediate implantation warrants further investigation. Large-scale randomized controlled trials are therefore imperative.

## Conclusion

This study demonstrates that robotic-assisted implant surgery significantly improves implant placement accuracy compared to traditional freehand methods. The innovative use of robotic technology, coupled with a robust comparative analysis and comprehensive evaluation metrics, underscores the potential of r-CAIS to enhance clinical outcomes in implant dentistry. These findings provide a strong foundation for future research and clinical application of robotic systems in dental surgery.

## Data Availability

The datasets used during the study are available from the corresponding author on reasonable request.

## Abbreviations

CBCT: Cone Beam Computed Tomography
DICOM: Digital Imaging and Communications in Medicine
CAIS: Computer-Assisted Implant Surgery
r-CAIS: Robotic Computer-Assisted Implant Surgery
s-CAIS: Static Computer-Assisted Implant Surgery
d-CAIS: Dynamic Computer-Assisted Implant Surgery

## References

[1] Derks J, Håkansson J, Wennström JL, Tomasi C, Larsson M, Berglundh T. Effectiveness of implant therapy analyzed in a Swedish population: early and late implant loss. J Dent Res. 2015;94:S44–51. 10.1177/0022034514563077 PMC4541089 25503901

[2] Kaewsiri D, Panmekiate S, Subbalekha K, Mattheos N, Pimkhaokham A. The accuracy of static vs. dynamic computer-assisted implant surgery in single tooth space: a randomized controlled trial. Clin Oral Implants Res. 2019;30:505–14. 31060099 10.1111/clr.13435

[3] Wismeijer D, Joda T, Flügge T, Fokas G, Tahmaseb A, Bechelli D et al. Group 5 ITI Consensus Report: Digital technologies. Clin Oral Implants Res [Internet]. 2018;29:436–42. Available from: https://onlinelibrary.wiley.com/doi/abs/10.1111/clr.13309 10.1111/clr.13309 30328201

[4] Chen YT, Chiu YW, Peng CY. Preservation of Inferior Alveolar Nerve Using the Dynamic Dental Implant Navigation System. J Oral Maxillofac Surg [Internet]. 2020;78:678–9. Available from: 10.1016/j.joms.2020.01.007 10.1016/j.joms.2020.01.007 32061618

[5] Aydemir CA, Arısan V. Accuracy of dental implant placement via dynamic navigation or the freehand method: a split-mouth randomized controlled clinical trial. Clin Oral Implants Res. 2020;31:255–63. 31829457 10.1111/clr.13563

[6] Casap N, Nadel S, Tarazi E, Weiss EI. Evaluation of a navigation system for dental implantation as a tool to train novice dental practitioners. J Oral Maxillofac Surg [Internet]. 2011;69:2548–56. Available from: 10.1016/j.joms.2011.04.026 10.1016/j.joms.2011.04.026 21821328

[7] Schnutenhaus S, Edelmann C, Knipper A, Luthardt RG. Accuracy of dynamic computer-assisted implant placement: a systematic review and meta-analysis of clinical and in vitro studies. J Clin Med. 2021;10:1–20. 10.3390/jcm10040704 PMC7916851 33670136

[8] D’haese J, Ackhurst J, Wismeijer D, De Bruyn H, Tahmaseb A. Current state of the art of computer-guided implant surgery. Periodontol. 2000. 2017. pp. 121–33. 10.1111/prd.12175 28000275

[9] Joda T, Derksen W, Wittneben JG, Kuehl S. Static computer-aided implant surgery (s-CAIS) analysing patient-reported outcome measures (PROMs), economics and surgical complications: a systematic review. Clin Oral Implants Res. 2018;29:359–73. 30328203 10.1111/clr.13136

[10] Raico Gallardo YN, da Silva-Olivio IRT, Mukai E, Morimoto S, Sesma N, Cordaro L. Accuracy comparison of guided surgery for dental implants according to the tissue of support: a systematic review and meta-analysis. Clin Oral Implants Res. 2017;28:602–12. 27062555 10.1111/clr.12841

[11] Pimkhaokham A, Jiaranuchart S, Kaboosaya B, Arunjaroensuk S, Subbalekha K, Mattheos N. Can computer-assisted implant surgery improve clinical outcomes and reduce the frequency and intensity of complications in implant dentistry? A critical review. Periodontol. 2000. John Wiley and Sons Inc; 2022. pp. 197–223. 10.1111/prd.12458 PMC9805105 35924457

[12] Lin CC, Wu CZ, Huang MS, Huang CF, Cheng HC, Wang DP. Fully digital workflow for planning static guided implant surgery: a prospective accuracy study. J Clin Med. 2020;9:1–15. 10.3390/jcm9040980 PMC7231012 32244735

[13] Tallarico M, Kim YJ, Cocchi F, Martinolli M, Meloni SM. Accuracy of newly developed sleeve-designed templates for insertion of dental implants: A prospective multicenters clinical trial. Clin Implant Dent Relat Res [Internet]. 2019 [cited 2023 Mar 27];21:108–13. Available from: https://onlinelibrary.wiley.com/doi/full/10.1111/cid.12704 10.1111/cid.12704 30592125

[14] Block M, Emery R, Lank K, Ryan J. Implant Placement Accuracy using dynamic Navigation. Int J Oral Maxillofac Implants. 2017;32:92–9. 27643585 10.11607/jomi.5004

[15] Panchal N, Mahmood L, Retana A, Emery R. Dynamic Navigation for Dental Implant Surgery. Oral Maxillofac Surg Clin North Am [Internet]. 2019;31:539–47. Available from: 10.1016/j.coms.2019.08.001 10.1016/j.coms.2019.08.001 31563194

[16] Wang W, Zhuang M, Li S, Shen Y, Lan R, Wu Y et al. Exploring training dental implant placement using static or dynamic devices among dental students. Eur J Dent Educ. 2022;438–48. 10.1111/eje.12825 35579548

[17] Ruppin J, Popovic A, Strauss M, Spüntrup E, Steiner A, Stoll C. Evaluation of the accuracy of three different computer-aided surgery systems in dental implantology: optical tracking vs. stereolithographic splint systems. Clin Oral Implants Res. 2008;19:709–16. 18492085 10.1111/j.1600-0501.2007.01430.x

[18] Vercruyssen M, Cox C, Coucke W, Naert I, Jacobs R, Quirynen M. A randomized clinical trial comparing guided implant surgery (bone- or mucosa-supported) with mental navigation or the use of a pilot-drill template. J Clin Periodontol. 2014;41:717–23. 24460748 10.1111/jcpe.12231

[19] Chen C-K, Yuh D-Y, Huang R-Y, Fu E, Tsai C-F, Chiang C-Y. Accuracy of Implant Placement with a Navigation System, a Laboratory Guide, and Freehand Drilling. Int J Oral Maxillofac Implants. 2018;33:1213–8. 30427951 10.11607/jomi.6585

[20] Schnutenhaus S, Wagner M, Edelmann C, Luthardt RG, Rudolph H. Factors influencing the accuracy of freehand implant placement: a prospective clinical study. Dent J. 2021;9:1–12. 10.3390/dj9050054 PMC8151810 34068734

[21] Wu Y, Wang F, Fan S, Chow JKF. Robotics in Dental Implantology. Oral Maxillofac Surg Clin North Am [Internet]. 2019;31:513–8. Available from: 10.1016/j.coms.2019.03.013 10.1016/j.coms.2019.03.013 31103316

[22] Bolding SL, Reebye UN. Accuracy of haptic robotic guidance of dental implant surgery for completely edentulous arches. J Prosthet Dent [Internet]. 2022;128:639–47. Available from: 10.1016/j.prosdent.2020.12.048 10.1016/j.prosdent.2020.12.048 33678441

[23] Feng Y, Fan J, Tao B, Wang S, Mo J, Wu Y et al. An image – guided hybrid robot system for dental implant surgery. Int J Comput Assist Radiol Surg [Internet]. 2021; Available from: 10.1007/s11548-021-02484-0 10.1007/s11548-021-02484-0 34449036

[24] Xu Z, Xiao Y, Zhou L, Lin Y, Su E, Chen J et al. Accuracy and efficiency of robotic dental implant surgery with different human-robot interactions: An in vitro study. J Dent [Internet]. 2023;137:104642. Available from: 10.1016/j.jdent.2023.104642 10.1016/j.jdent.2023.104642 37517786

[25] Bai SZ, Ren N, Feng ZH, Xie R, Dong Y, Li ZW, et al. [Animal experiment on the accuracy of the Autonomous Dental Implant Robotic System]. Zhonghua Kou Qiang Yi Xue Za Zhi = Zhonghua Kouqiang Yixue zazhi = Chinese. J Stomatol. 2021;56:170–4. 10.3760/cma.j.cn112144-20210107-00008 33557501

[26] Yang S, Chen J, Li A, Deng K, Li P, Xu S. Accuracy of autonomous robotic surgery for single-tooth implant placement: a case series. J Dent. 2023;132:1–8. 10.1016/j.jdent.2023.104451 36781099

[27] Li Y, Hu J, Tao B, Yu D, Shen Y, Fan S et al. Automatic robot-world calibration in an optical-navigated surgical robot system and its application for oral implant placement. Int J Comput Assist Radiol Surg [Internet]. 2020;15:1685–92. Available from: 10.1007/s11548-020-02232-w 10.1007/s11548-020-02232-w 32715383

[28] Chen J, Zhuang M, Tao B, Wu Y, Ye L, Wang F. Accuracy of immediate dental implant placement with task-autonomous robotic system and navigation system: an in vitro study. Clin Oral Implants Res. 2023;1–11. 10.1111/clr.14104 37248610

[29] Tao B, Feng Y, Fan X, Zhuang M, Chen X, Wang F et al. Accuracy of dental implant surgery using dynamic navigation and robotic systems: An in vitro study. J Dent [Internet]. 2022;123:104170. Available from: 10.1016/j.jdent.2022.104170 10.1016/j.jdent.2022.104170 35679989

[30] Chen J, Bai X, Ding Y, Shen L, Sun X, Cao R, et al. Comparison the accuracy of a novel implant robot surgery and dynamic navigation system in dental implant surgery: an in vitro pilot study. BMC Oral Health. 2023;23:1–9. 36978064 10.1186/s12903-023-02873-8 PMC10052843

[31] Jia S, Wang G, Zhao Y, Wang X. Accuracy of an autonomous dental implant robotic system versus static guide-assisted implant surgery: A retrospective clinical study. J Prosthet Dent [Internet]. 2023;1–9. Available from: 10.1016/j.prosdent.2023.04.027 10.1016/j.prosdent.2023.04.027 37291043

[32] Chen W, Al-Taezi KA, Chu CH, Shen Y, Wu J, Cai K et al. Accuracy of dental implant placement with a robotic system in partially edentulous patients: a prospective, single-arm clinical trial. Clin Oral Implants Res. 2023;707–18. 10.1111/clr.14083 37167364

[33] Sailer I, Karasan D, Todorovic A, Ligoutsikou M, Pjetursson BE. Prosthetic failures in dental implant therapy. Periodontol 2000. 2022;88:130–44. 35103329 10.1111/prd.12416 PMC9305548

[34] Franchina A, Stefanelli LV, Maltese F, Mandelaris GA, Vantaggiato A, Pagliarulo M et al. Validation of an intra-oral scan method versus cone beam computed tomography superimposition to assess the accuracy between planned and achieved dental implants: A randomized in vitro study. Int J Environ Res Public Health [Internet]. 2020;17:1–21. Available from: 10.1016/j.joms.2017.02.026 10.3390/ijerph17249358 PMC7765074 33542168

[35] Tahmaseb A, Wu V, Wismeijer D, Coucke W, Evans C. The accuracy of static computer-aided implant surgery: a systematic review and meta-analysis. Clin Oral Implants Res. 2018;29:416–35. 30328191 10.1111/clr.13346

[36] Block MS. Accuracy using static or dynamic navigation. J Oral Maxillofac Surg. 2016;74:2–3. 27110617 10.1016/j.joms.2015.11.002

[37] Sittikornpaiboon P, Arunjaroensuk S, Kaboosaya B, Subbalekha K, Mattheos N, Pimkhaokham A. Comparison of the accuracy of implant placement using different drilling systems for static computer-assisted implant surgery: A simulation-based experimental study. Clin Implant Dent Relat Res [Internet]. 2021;23:635–43. Available from: https://onlinelibrary.wiley.com/doi/abs/10.1111/cid.13032 10.1111/cid.13032 34288341

[38] El Kholy K, Janner SFM, Schimmel M, Buser D. The influence of guided sleeve height, drilling distance, and drilling key length on the accuracy of static computer-assisted Implant surgery. Clin Implant Dent Relat Res. 2019;21:101–7. 30589502 10.1111/cid.12705

[39] Golob Deeb J, Bencharit S, Carrico CK, Lukic M, Hawkins D, Rener-Sitar K, et al. Exploring training dental implant placement using computer-guided implant navigation system for predoctoral students: a pilot study. Eur J Dent Educ. 2019;23:415–23. 31141291 10.1111/eje.12447

[40] Sun TM, Lee HE, Lan TH. The influence of dental experience on a dental implant navigation system. BMC Oral Health. 2019;19:1–12. 31623636 10.1186/s12903-019-0914-2 PMC6798373

[41] Somogyi-Ganss E, Holmes HI, Jokstad A. Accuracy of a novel prototype dynamic computer-assisted surgery system. Clin Oral Implants Res. 2015;26:882–90. 24837492 10.1111/clr.12414

[42] Schneider D, Marquardt P, Zwahlen M, Jung RE. A systematic review on the accuracy and the clinical outcome of computer-guided template-based implant dentistry. Clin Oral Implants Res [Internet]. 2009;20:73–86. Available from: https://onlinelibrary.wiley.com/doi/abs/10.1111/j.1600-0501.2009.01788.x 10.1111/j.1600-0501.2009.01788.x 19663953

[43] Van Assche N, Vercruyssen M, Coucke W, Teughels W, Jacobs R, Quirynen M. Accuracy of computer-aided implant placement. Clin Oral Implants Res [Internet]. 2012;23:112–23. Available from: https://onlinelibrary.wiley.com/doi/abs/10.1111/j.1600-0501.2012.02552.x 10.1111/j.1600-0501.2012.02552.x 23062136

[44] Jorba-García A, González-Barnadas A, Camps-Font O, Figueiredo R, Valmaseda-Castellón E. Accuracy assessment of dynamic computer–aided implant placement: a systematic review and meta-analysis. Clin Oral Investig. 2021;25:2479–94. 33635397 10.1007/s00784-021-03833-8

[45] Pellegrino G, Ferri A, Del Fabbro M, Prati C, Gandolfi MG, Marchetti C. Dynamic Navigation in Implant Dentistry: a systematic review and Meta-analysis. Int J Oral Maxillofac Implants. 2021;36:e121–40. 34698720 10.11607/jomi.8770

[46] Cheng K, Kan T, Liu Y, Zhu W, Zhu F, Wang W et al. Accuracy of dental implant surgery with robotic position feedback and registration algorithm: An in-vitro study. Comput Biol Med [Internet]. 2021;129:104153. Available from: https://www.sciencedirect.com/science/article/pii/S0010482520304844 10.1016/j.compbiomed.2020.104153 33260102

[47] Yang G-Z, Cambias J, Cleary K, Daimler E, Drake J, Dupont PE et al. Medical robotics—Regulatory, ethical, and legal considerations for increasing levels of autonomy. Sci Robot [Internet]. 2017;2:eaam8638. Available from: https://www.science.org/doi/abs/10.1126/scirobotics.aam8638 10.1126/scirobotics.aam8638 33157870

[48] Putra RH, Yoda N, Astuti ER, Sasaki K. The accuracy of implant placement with computer-guided surgery in partially edentulous patients and possible influencing factors: a systematic review and meta-analysis. J Prosthodont Res. 2022;66:29–39. 33504723 10.2186/jpr.JPR_D_20_00184

